# *QuickDeconvolution*: fast and scalable deconvolution of linked-read sequencing data

**DOI:** 10.1093/bioadv/vbac068

**Published:** 2022-09-26

**Authors:** Roland Faure, Dominique Lavenier

**Affiliations:** INRIA RBA, CNRS UMR 6074, University of Rennes, Rennes, France; INRIA RBA, CNRS UMR 6074, University of Rennes, Rennes, France

## Abstract

**Motivation:**

Recently introduced, linked-read technologies, such as the 10× chromium system, use microfluidics to tag multiple short reads from the same long fragment (50–200 kb) with a small sequence, called a *barcode*. They are inexpensive and easy to prepare, combining the accuracy of short-read sequencing with the long-range information of barcodes. The same barcode can be used for several different fragments, which complicates the analyses.

**Results:**

We present *QuickDeconvolution* (QD), a new software for *deconvolving* a set of reads sharing a barcode, i.e. separating the reads from the different fragments. QD only takes sequencing data as input, without the need for a reference genome. We show that QD outperforms existing software in terms of accuracy, speed and scalability, making it capable of deconvolving previously inaccessible data sets. In particular, we demonstrate here the first example in the literature of a successfully deconvoluted animal sequencing dataset, a 33-Gb *Drosophila melanogaster* dataset. We show that the taxonomic assignment of linked reads can be improved by deconvoluting reads with QD before taxonomic classification.

**Availability and implementation:**

Code and instructions are available on https://github.com/RolandFaure/QuickDeconvolution.

**Supplementary information:**

Supplementary data are available at *Bioinformatics Advances* online.

## 1. Introduction

### 1.1 Linked-read sequencing

Since the discovery of the role of DNA in the transmission of genetic information (Avery *et al.*, 1944), it has been understood that obtaining the genomes of organisms is essential for understanding their biology. Thus, great efforts have been made to extract genomic information from a variety of organisms, including humans (International Human Genome Sequencing Consortium, 2001).

The first modern sequencers, with which scientists were able to recover the precise sequence of (small) strands of DNA called *reads*, appeared around 1970 and were named after Sanger, the scientist who created the technique (Sanger *et al.*, 1977). Since then, a wide variety of techniques have been proposed. Those still in use can be classified into two broad categories: (i) short-read sequencers, which are capable of producing a large quantity of short reads (<300 bp) with a very low error rate (usually <1%) and low cost; (ii) long-read sequencers, which are capable of producing much longer reads [more than 10 kb and up to 2 million bp in extreme cases (Payne *et al.*, 2019)] but with a generally much higher error rate; the samples are also considerably more difficult to prepare.

Linked-read technologies were developed as a compromise between short, accurate reads and long, inaccurate reads; 10× sequencing is its oldest and most common form, but today a variety of new techniques are emerging, such as LoopSeq, TELL-Seq and BGI long fragment reads. To produce linked reads, long DNA fragments are separated and sequenced with short reads. Short reads typically cover 10–20% of the fragment length. A ’barcode’ is attached to the end of each read in the form of a small DNA sequence. All reads from the same fragment share the same barcode.

### 1.2 The barcode deconvolution problem

Using the terminology defined in previous papers (Danko *et al.*, 2019; Mak *et al.*, 2021), the set of reads sharing the same barcode will be referred to as a *read cloud*. The barcodes provide implicit long-range information: two reads sharing the same barcode originate with high probability from the same fragment and are thus ‘not far away’ on the DNA strand. This long-range information can be exploited by appropriate software while being much cheaper and easier to prepare than long-read sequencing (Wang *et al.*, 2019). Typically, linked reads can be used to phase haplotypes (Zheng *et al.*, 2016) or to propose better *de novo* genome assemblies. For instance, a reference for the pepper genome was provided in 2018 using linked reads (Hulse-Kemp *et al.*, 2018).

A new computing challenge arising from these technologies is that the total number of barcodes is limited. 10× sequencing technology, for example, provides only a few million barcodes. Because the total number of fragments routinely exceeds this number, barcodes must be used multiple times for many different fragments. This complicates the exploitation of the data. The barcode deconvolution problem can be defined as the separation of the reads of the different fragments present in each barcode. The ultimate goal is to obtain ‘enhanced barcodes’, where each barcode identifies only the reads of a single DNA fragment. Downstream methods are then much more efficient than raw barcodes, as shown by Shajii *et al.* (2018).

### 1.3 State of the art

Two main approaches can be used to deconvolve a set of barcoded reads: reference-based or reference-free approaches.

When the content of the sequencing experiment is roughly known in advance, and reference genomes are available, for example when sequencing a model organism such as Human or Drosophila, a reference-based approach can be used with the EMA software (Shajii *et al.*, 2018). EMA maps all reads from each read cloud to a reference genome (or several in the case of metagenomic data). Since reads from the same fragment are close to each other on the sequenced genome(s), there is a good probability that they will also map close to each other on the reference genome(s). Although they often provide good results, reference-based approaches are not always possible or desirable. Good quality references are not always available and sometimes the species in the sample are not known in advance. Moreover, the result will be biased by the reference genome(s): using two different references may give two different solutions. In this article, we present an approach without reference, free of these biases.

The first reference-free barcode deconvolution software was published in Danko *et al.* (2019), under the name *Minerva*. It uses the fact that samples are sequenced with some *coverage*, i.e. that all portions of the genome are sequenced multiple times, usually more than 20 times. Many fragments with different barcodes will therefore come from the same region, which Minerva can exploit. The principle of Minerva is the same as that of our software, *QuickDeconvolution* (QD), and will be deeply discussed later. The paper established a strong theoretical foundation for the method and showed its application on two sets of mock metagenomes. However, the method remained too slow to be used on large or even medium-sized datasets and is referred to by its authors as a ‘proof of concept’ algorithm.

Very recently, another reference-free software from the authors of Minerva has been proposed under the name *Ariadne* (Mak *et al.*, 2021). Based on a totally different concept, Ariadne starts by doing a complete assembly of the de-Bruijn graph of reads using the SPAdes assembler (Prjibelski *et al.*, 2020), ignoring the barcodes. It then proceeds barcode by barcode. The key idea is that two reads coming from the same fragment must not be far from each other on the assembly graph. Ariadne therefore considers that if two reads sharing the same barcode are close on the assembly graph, then they come from the same fragment. Minerva and Ariadne were only proven to be capable of deconvolving metagenomic datasets.

### 1.4 Contribution

We present QD, a reference-free software to solve the problem of barcode deconvolution. QD takes barcodes in fastq format as input and produces an enhanced fastq file, where the barcodes are marked with an additional number indicating the subgroups in the read cloud.

Based on the same principle as Minerva, QD brings two crucial improvements: (1) an optimized algorithm with parallel implementation; (2) an additional clustering step, offering better accuracy.

We show that QD outperforms other reference-free barcode deconvolution software in both speed and accuracy. By lifting some resource limitations, QD can deconvolve previously intractable datasets. The higher accuracy of the algorithm allows the program to deconvolve single-species repeat-rich datasets. Here, we provide the first example in the literature of a deconvoluted animal dataset, from the species *Drosophila melanogaster*. The availability of reference genomes allowed us to confirm the quality of the deconvolutions proposed by QD.

## 2. Algorithm

### 2.1 Principle

The basic principle of the QD algorithm is the same as that of Minerva (Danko *et al.*, 2019). It is based on the fact that all regions of the genome are cloned during sequencing and will be sequenced multiple times: many fragments will therefore come from the same region and share part of their sequence. If two fragments share the same sequence over part of their length, they are called *overlapping*. Minerva and QD make use of the fact that several overlapping fragment reads will probably overlap. In other words, multiple reads of a fragment will likely overlap with multiple reads of an overlapping fragment with a different barcode. The fragments of a barcode can then be distinguished by the set of barcodes they overlap.

**Fig. 1.**
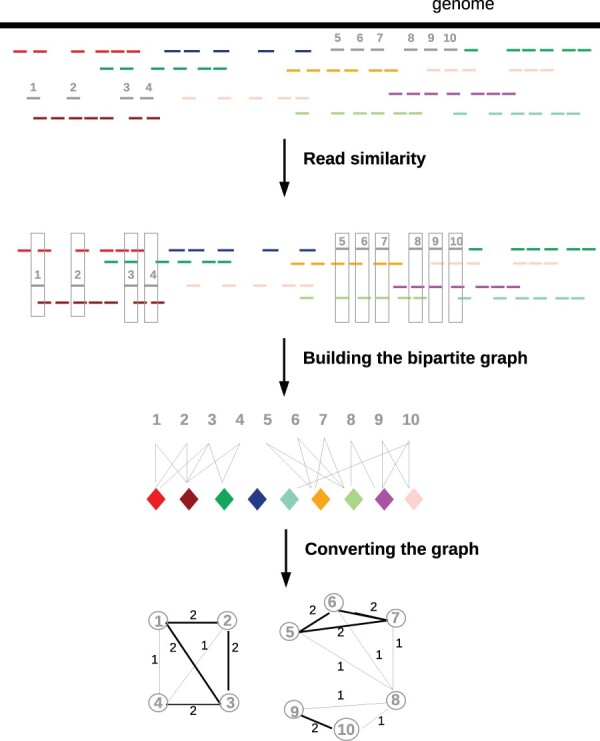
Illustration of the principle behind QD and Minerva. Top: from the genome, reads are sequenced and barcoded. Barcodes are represented as colors. The 10 reads of the grey cloud, that actually come from two different fragments (1234 and 5 678 910), will be deconvolved. Each grey read is compared to all the other reads of the dataset. A bipartite graph is built, linking each read to all the barcodes it overlaps. It is then converted to a graph between all the reads from the barcode. The strength of a link is the number of shared barcodes the two reads overlap. Minerva then outputs as result all the connected components of the graph, while QD clusters the graph

More precisely, each barcode is processed separately. For each barcode, let’s call it *anchor*, a bipartite graph is constructed, with all the anchor reads on one side, and all the barcodes of the experiment on the other side. For each read from the anchor, the set of all overlapping reads (with an overlap of ≥k bp) in the sequencing data is found. Links are added in the graph between each read and the individual barcodes of its overlapping reads. Once all the anchor reads are processed, the graph is complete. The bipartite graph is then converted into a graph containing only the reads of the anchor: two reads are linked by a link of strength *n* if they overlap with *n* shared barcodes. Since reads from the same fragment tend to overlap with the same barcodes, it is expected that this graph can then be clustered, with each cluster containing reads from a single fragment. This algorithm is illustrated in Figure 1.

### 2.2 Mathematical justification

The mathematical justification of the model has been very well described in Danko *et al.* (2019) for metagenomic samples. We will propose thereafter a natural extension in the case of a multi-chromosomic genome. Let us justify the key assumption that, within a barcode, two reads from the same fragment likely overlap more shared barcodes than two reads from two different fragments.

Let us consider that fragments of length *L* are drawn with a uniform probability across the genome. Let *p* be the proportion of each fragment covered by reads, typically 10–20% in the case of 10× data. Enough fragments are drawn to cover the genome with read coverage *c* (thus fragment coverage =c/p). In total, *N_tot_* different barcodes are attached, with on average *n* molecules per barcode.

Two reads from the same fragment distant by *l *<* L* on the strand overlap on average with

$$p_{1}=\frac{L-l}{L}*(\frac{c}{p}-1)$$

common fragments. Let *P* be the probability that a read from a common overlapping fragment overlaps one of the two reads. *P*^2^ is approximately the probability that two reads from the common fragment overlap the two reads. Consequently, two reads from the same fragment overlap on average $p_{1}*P^{2}$ shared barcodes.

Two reads with the same barcode but from two far away fragments overlap on average

$$p_{2}=\frac{c}{p}*[\frac{c}{p}*\frac{n-1}{n*N_{\mathrm{tot}}-\frac{c}{p}}]$$

common fragments. Indeed, the first read overlaps on average with $\frac{c}{p}$ fragments, thus with roughly $\frac{c}{p}$ different barcodes (let these barcodes be the *read1-barcodes*). All other fragments are now called the *far-away fragments*. The total number of far-away fragments with a given read1-barcode attached is on average *n −* 1. The total number of far-away fragments is $n*N_{\mathrm{tot}}-\frac{c}{p}$. The second read overlaps with $\frac{c}{p}$ fragments, to which roughly $\frac{c}{p}$ different *read2-barcodes* are attached. The probability of a given read1-barcode being also a read2-barcode is $\frac{c}{p}*\frac{n-1}{n*N_{\mathrm{tot}}-\frac{c}{p}}$. Consequently, the two reads overlap on average with $p_{2}*P^{2}$ shared barcodes.

In linked read experiments, the order of magnitude of *N_tot_* is at least 10^6^, while the order of magnitude of *c*/*p* is at most 10^3^. Consequently, $p_{1}\gg p_{2}$: two reads close on the same fragment will overlap on average many more shared barcodes than two reads from two different fragments.

While the estimated link strengths in this model tend to ensure very reliable linkages, it is important to keep in mind that in our model, an overlap between two reads means that both reads come from the same region. This is usually a false assumption, as repeated regions are common in genomic data and will cause many artifactual links between reads. The graph should therefore be handled with care.

### 2.3 Read similarity

For each barcode, all reads are processed iteratively. For each read, the set of all overlapping reads in the dataset must be found. Since there are millions of fragments in the dataset, finding this set is one of the key difficulties of the program. The problem is well known in the genome assembly community, where many overlapping reads must be assembled into longer DNA sequences (Li *et al.*, 2012). The strategy implemented by QD is a well-known strategy based on *k-mers*.

k-mers are subsequences of length *k* present in the reads. In a preliminary indexing phase, a dictionary is constructed, reporting for the k-mers in the dataset the list of all their occurrences in the reads. In our experiments, the value of *k* has been set to 20.

To speed up the indexing process, QD indexes at first sight only a user-defined fraction of k-mers *d*. A k-mer is indexed if it is found among the smallest *d* fraction of all possible k-mers by lexicographical order. For example, if *d *=* *0.25, all the k-mers starting with ‘A’ will be indexed. If no k-mers are indexed in a window of size *w* (user-specified), k-mers among the smallest 2*d fraction of k-mers are indexed in this window. If still no k-mers are found in the window, k-mers among the smallest 3*d fraction of k-mers are indexed there, etc. This ensures the fundamental property that all stretches of length *w* on any read contain at least one indexed k-mer, and thus that two reads overlapping by *w* bases will share at least one indexed k-mer, even in highly GC-biased regions. On our tests, the precision of the deconvolution started decreasing when *d* went below 1/8.

To each read is attached the set of its indexed k-mers. To maximize the speed of execution and avoid the costly process of alignment, QD never checks if two reads overlap. It goes through the set of indexed k-mers of a read and finds in the index all other reads containing these k-mers. Two reads sharing at least three indexed k-mers are flagged as ‘similar’.

### 2.4 Graph building

For each barcode, a graph linking all the reads in the cloud is constructed, as described above: first, a bipartite graph between the reads in the cloud and the barcodes is built, and then it is converted into a graph containing only the reads in the cloud. Two reads are linked if they respectively overlap with two reads in the dataset that have identical barcodes. As demonstrated above with a simple mathematical model, two reads from the same fragment will be linked with a much higher probability than two reads from two different fragments.

Tests on single-species datasets show that repeated regions can create false positive links in the graph. This is because a read containing a repeated region will share k-mers with all reads containing that repeated region, including all those that are actually far away on the genome. Repeated k-mers are present in many more reads than average k-mers, creating hubs of connections and many false positive links in the graph. We observed that de-indexing k-mers present many more times than the average number of times in the dataset greatly improves the quality of deconvolution on repeat-rich datasets, while not affecting deconvolution on repeat-poor datasets. The best results were obtained by de-indexing k-mers present twice the average number of times or more. The disadvantage is that some reads are not indexed at all, especially in repeated regions, although this is mitigated by the fact that only one of the two reads in the pair needs to be indexed to be deconvoluted. The worst-case scenario for assembly would be that multiple fragments with the same barcode lie within large segmental duplications and thus could not be distinguished. The effect of de-indexing reads is evaluated (Supplementary Fig. S1), showing that this procedure is necessary for the repeat-rich dataset we tested.

### 2.5 Graph clustering

The read graph must then be clustered into an unknown number of enhanced read clouds. QD uses the *Chinese whispers* algorithm, a clustering method introduced in NLP search (Biemann, 2006). It works as follows: at the beginning of the algorithm, each read is contained in its own cluster of size 1. The reads are then processed in a random order until convergence. Each read inherits the cluster that is seen most often among all neighbors, weighted by the strengths of the links (in case of several equal possibilities, one is chosen randomly). This algorithm is known to converge quickly to a few stable clusters, especially if the diameter of the graph is small (i.e. any two vertices are separated by few edges), which is usually the case in our read graphs. In the worst case, the clustering can oscillate, but this is marginal in practice for QD.

This clustering method has the great advantage of being parameter-free and agnostic regarding the final number of clusters.

This graph clustering method is a novelty compared to what was done in Minerva. Minerva removes links weaker than a certain threshold on the read graph and then considers the different connected components of the graph as separate clouds. Danko *et al.* showed that the method could work to separate fragments from different species in metagenomic samples. However, when trying to separate fragments from a single genome, we found that it was difficult to fully separate clusters due to redundancies and small repeated elements present across the genome. It became increasingly difficult as sequencing depth or read quality decreased. Therefore, we opted for a slightly more expensive but more flexible approach that allows for residual false positives. Knowing that the clustering step will compensate for some errors, we could implement shortcuts to make the graph construction step faster.

Finally, all reads in a cloud that are not related to the graph are not grouped separately but are marked with a special tag ‘0’, to indicate to that clustering was ineffective at that location. This can happen when a read is too noisy to be overlapped with anything else, or if the read is in a highly repeated region and all of its k-mers are not indexed.

### 2.6 Parallelization

An imperative for accelerating QD is to parallelize the program. The goal of parallelization is to distribute the work among the different threads present in a compute node. A perfectly parallelized program is able to run *t* times faster when *t* threads are available.

The algorithm runs in four distinct phases: loading the data from the input file, creating the dictionary, deconvolving the read clouds and writing the data to an output file. The first and last phases being negligible in time compared to the other two, they are not parallelized at all and processed by a single thread.

The third phase, where each graph is built and clustered, is trivially parallelizable. Threads can manage separate clouds: build their graphs, cluster them and store the result. Threads compete only for access to the dictionary, which is not copied *t* times to keep RAM usage reasonable.

The construction of the dictionary is the most difficult phase to parallelize. Indeed, the threads cannot simply distribute the reads between them: if the same k-mer is found on two threads at the same time, the threads cannot update the dictionary simultaneously (if two threads write the same entry at the same time, an entry will probably be overwritten). If the k-mer has not been seen before, this can even crash the program. The trick is to divide the k-mers between the threads: for example, thread 1 takes care of k-mers ending in A or C while thread 2 takes care of k-mers ending in G and T. Each thread must examine all reads but does not index all k-mers. To avoid recalculating in each thread the set of sparse k-mers, this calculation is done beforehand for all reads, in parallel. We end up with two sub-phases: first, the threads distribute the reads among themselves and compute all the sparse k-mers; once this is done, the threads distribute the k-mers among themselves and go through all the reads to index them.

## 3. Datasets and evaluation metrics

### 3.1 Datasets

QD was benchmarked on five datasets.

The first one is a simulated dataset based on the genome of *Escherichia coli*. To introduce a little complexity and because linked reads have often been used to phase haplotypes, we created a ‘fake diploid’ *E.coli* by duplicating the genome and introducing a 1% difference between the two chromosomes. To simulate 10× sequencing, fragments of 70–130 kb were drawn uniformly along the genome. 15% of the length of each fragment was covered by paired-end 150 bp reads with 1% error. Barcodes were then randomly assigned to all these fragments. Enough fragments were drawn to obtain a final read coverage of 50. The total number of barcodes available was computed to be four times less than the total number of fragments. That resulted in a 0.6-Gb dataset.

We also created a simulated *Homo sapiens* dataset. 10× sequencing was simulated using LRSim (Luo *et al.*, 2017), a linked-read simulator built to reproduce biases and errors of linked-read sequencing. We chose to run the simulator over chromosome 1 of the genome of *H.sapiens*. The sequenced dataset is 7 Gb.

The deconvolution software was also tested on the sequencing of two metagenomic mock communities (i.e. communities where the mix of species is precisely known). The first one was a 10× sequencing run on the metagenomic sample MSA1003, a mix of 10 species sold by the ATCC company. It resulted in 108 Gb of data, published in the paper (Zhang *et al.*, 2020). The second one was a LoopSeq sequencing run on a discontinued ATCC mix of five species. The size of the dataset is 9 Gb. To measure how well the software deconvolved the reads, the solution of the deconvolution was approximated with an approach similar to EMA: all the reads were mapped to the set of reference genomes using Bowtie2. Reads that had the same barcode and mapped <100 kb away on the same genome were considered as coming from the same fragment.

The last dataset comes from the 10× sequencing of a single animal species, *D.melanogaster*. It totaled 33Gb of sequencing data. The quality of deconvolution was assessed by the same method as with the metagenomic ATCC datasets. Seventy-four percent of the reads mapped uniquely on the reference genome, and overall 60% of the reads were identified to their fragment of origin with good confidence. Only the deconvolution of those reads was evaluated.

### 3.2 Evaluation metrics

In this section, a ‘reference cloud’ will refer to a set of reads coming from a single fragment according to the reference solution and ‘deconvolved cloud’ will refer to a set of reads proposed by a software as coming from one fragment.

To evaluate the deconvolution, two metrics are proposed, a classical approach for clustering evaluation. Indeed, good deconvolution is a compromise between two extremes. On one hand, all reads from a reference cloud must be kept together in the same deconvolved cloud (otherwise the reads are *over-deconvoluted*). On the other hand, all reads from a deconvoluted cloud must come from a single reference cloud (otherwise there are still several fragments per cloud, the reads are *under-deconvoluted*). We thus propose:

- The over-deconvolution entropy. It evaluates the disorder within each reference cloud by using the classical entropy formula $-\sum_{i}p_{i}*\log(p_{i})$, where *p_i_* is the proportion of reads of the reference cloud contained in deconvolved cloud *i*. If all reads of the reference cloud have been kept together in one deconvolved cloud, the over-deconvolution entropy is zero.
- The under-deconvolution entropy. It evaluates the disorder within each deconvolved cloud by using the classical entropy formula $-\sum_{j}p_{j}*\log(p_{j})$, where *p_j_* is the proportion of reads of the deconvolved cloud contained in reference cloud *j*. If the deconvolution is complete, and the deconvolved clouds contain only reads coming from one reference cloud, the under-deconvolution entropy is zero.

**Fig. 2.**
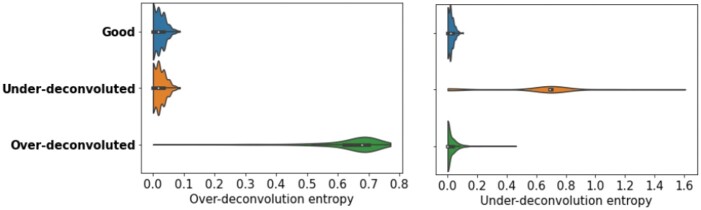
Illustration of over-deconvolution and under-deconvolution entropies on three mock deconvolutions. The ‘good’ deconvolution corresponds to a slightly noisy perfect deconvolution. In the ‘under-deconvoluted’ solution, two reference clouds were assigned to each improved barcode (instead of one). In the ‘over-deconvoluted’ solution each reference cloud was split between two improved barcodes.

A histogram of the over-deconvolution entropies of all reference clouds and of the under-deconvolution of all deconvolved clouds is drawn, the aim being to concentrate the distributions around zero. Quite logically, non-deconvoluted data (where the clouds just correspond to original barcodes) is under-deconvoluted but not over-deconvoluted. A graphical example on mock datasets is provided Figure 2.

## 4. Results

All results were obtained by running all software on a server housing 16 Intel Xeon CPUs with four cores each, running at 2.7 GHz. 3.1 TB of RAM was available.

QD, Minerva and Ariadne were run on the five datasets. Minerva and Ariadne were run with parameters proposed on GitHub. QD was run using *k* = 20 and indexing 1 over 8 k-mer on average.

The *Drosophila*, human and ATCC 10× datasets were too big for Minerva, which was killed after running 15 days. We were unable to run Ariadne on these three datasets because it generated huge intermediary files (≥12T), saturating the space available.

On *E.coli* dataset, Minerva proposed an enhanced barcode (sometimes identical to the original barcode) for only 1.4% of the reads, Ariadne for 69% and QD more than 99.9%. The most probable explanation for the low rate of reads deconvolved by Minerva is that the clusters of reads were slightly inter-connected, so Minerva could not deconvolve those without a clustering step.

On the ATCC loopseq dataset, Minerva proposed a deconvolution for <0.05% of the reads, as already reported in Mak *et al.* (2021) and was thus not evaluated. Ariadne and QD classified more than 99% of the reads.

QD proposed a deconvolution for 81% of the reads for *Drosophila*, 94% of the reads for ATCC 10× and more than 99.9% for *H.sapiens*.

Only the deconvolved reads have been taken into account to measure the quality of the clustering for each method.

### 4.1 Accuracy

**Fig. 3.**
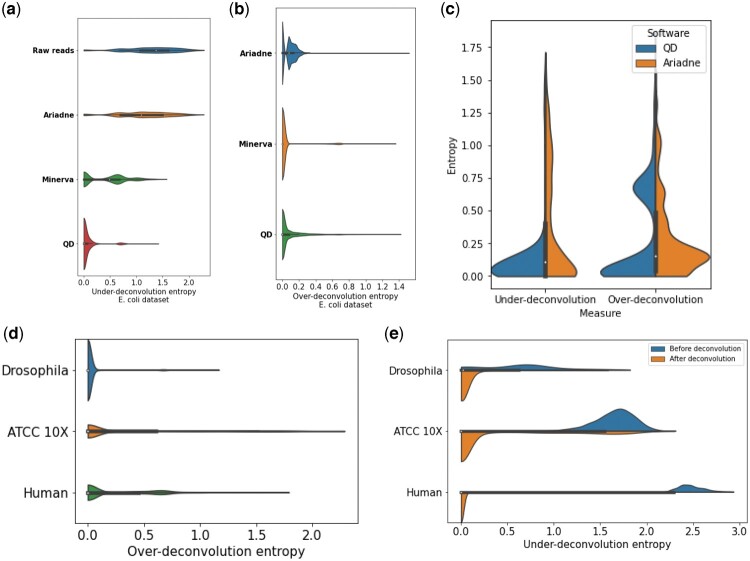
Evaluations of the quality of the deconvolution on the different datasets with different software. (**a**) Under-deconvolution entropy of the deconvolved *E.coli* dataset using different software. (**b**) Over-deconvolution entropy of the deconvolved *E.coli* dataset using different software. (**c**) Over- and under-deconvolution entropies of the ATCC Loopseq dataset after deconvolution with Ariadne and QD. (**d**) Over-deconvolution by QD of three datasets. (**e**) Under-deconvolution of datasets before and after being deconvoluted by QD

In terms of deconvolution, QD proves superior to the other tools. Figure 3a and e shows that deconvolution with QD greatly improves the under-deconvolution entropies. Minerva shows comparable performance on *E.coli*, and Ariadne on *ATCC Loopseq*. Ariadne, however, hardly improved the deconvolution of the raw reads of *E.coli*. This improvement might be due to the fact that the assembly graph of a mix of two strains is very tangled. Thus, many regions far away on the genome are close on the assembly graph and cannot be separated by Ariadne.

Figure 3(b–d) shows that in all deconvolutions proposed by Ariadne and QD a non-negligible number of deconvolved read clouds are slightly over-deconvoluted, i.e. have a few missing reads. For QD, they represent generally two or three reads that have been clustered separately from the rest of the cloud. In the human and loopseq cases (Fig. 3d and c), there is an over-deconvolution peak around an entropy of 0.7. It corresponds to reads from the same fragment split in two clouds of roughly equal size, corresponding to the two ends of the fragment. The resulting clouds remain nevertheless valid, in the sense that all reads within each cloud are actually close to each other on the genome.

### 4.2 Performance

**Fig. 4.**
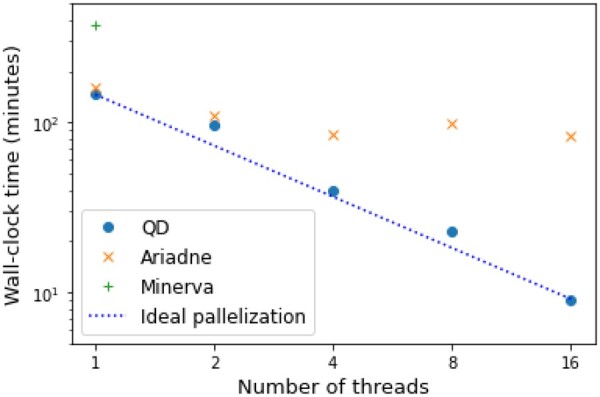
Run time (in minutes) of the different deconvolution algorithms on the *E.coli* dataset. Axes have logarithmic scales. The dashed black line represents the expected speed of QD if the parallelization was ideal

Speed was put forward by the authors of Minerva as the main limitation of their algorithm. Hence, it was one of the main focuses when developing QD. Run time of the different algorithms was measured using the command *time* of Linux system. We compared the algorithms mainly on the *E.coli* dataset. The results are plotted in Figure 4. The program has been run with 1, 2, 3, 8 and 16 threads.

**Fig. 5.**
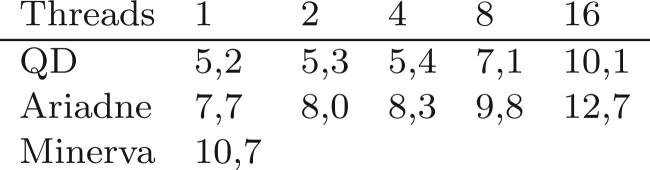
RAM usage (in gigabytes) of the different deconvolution software while deconvolving the *E.coli* dataset

For one or two threads, QD and Ariadne have nearly identical run tim, and both run roughly twice as fast as Minerva. When increasing the number of threads the run time of QD decreases almost ideally, at least up to 16 threads. Ariadne does not scale well, since it is only twice as fast with four threads and does not seem to accelerate at all beyond. We end up with an order of magnitude of difference in run time when running Ariadne and QD with 16 threads. RAM usage was comparable across the tools (Figure 5).

**Fig. 6.**
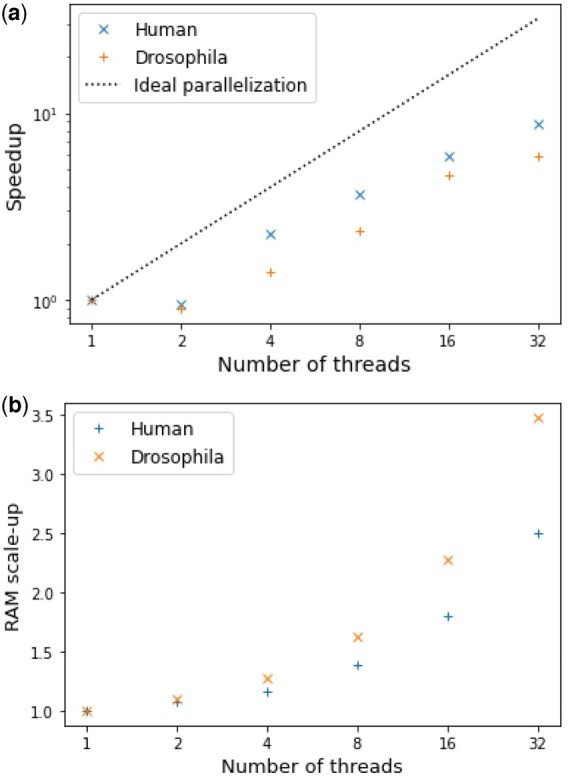
Behavior of multithreaded QD on the human and Drosophila datasets. (**a**) Speed-up of QD on the human and Drosophila datasets. Speed-up is defined as reference run time over run time. (**b**) Scale-up of the RAM usage of QD on the human and Drosophila datasets. Scale-up is defined as reference RAM usage over RAM usage

We conducted the further investigation on the effect of parallelization on the human and *Drosophila* datasets. Figure 6a is a plot of the speed-up of QD, i.e. the acceleration compared with the single-thread reference time. For QD, the parallelization becomes less interesting beyond 16 threads. This is an expected behavior: as threads begin to compete for memory access, parallelization becomes less interesting.

RAM usage was significant: for the *Drosophila* dataset, the RAM usage ranged from 459 to 1053 GB, while it ranged from 88 to 158 GB for the *H.sapiens* dataset. RAM usage tends to increase with the number of threads, even though all threads use a common memory space and that theoretically no extra information is stored. The *scale-up* of memory space used by QD is plotted in Figure 6b, showing the increase of RAM used with multiple threads compared to the reference single-threaded QD algorithm.

### 4.3 Application

Read clouds can be used to improve the taxonomic assignment of short reads. When classifying a set of short reads, many of them cannot be assigned to a low taxonomic rank. However, all reads in a fragment are from the same organism. This can help promote reads to lower ranks: a read can be promoted to a lower taxonomic rank that contains reads with the same barcode, provided there are no conflicts between multiple lower ranks. Conflicts occur when multiple fragments of closely related species have the same barcode. Deconvoluted read clouds reduce the probability of having conflicts between multiple ranks, thus improving taxonomic assignment.

**Fig. 7.**
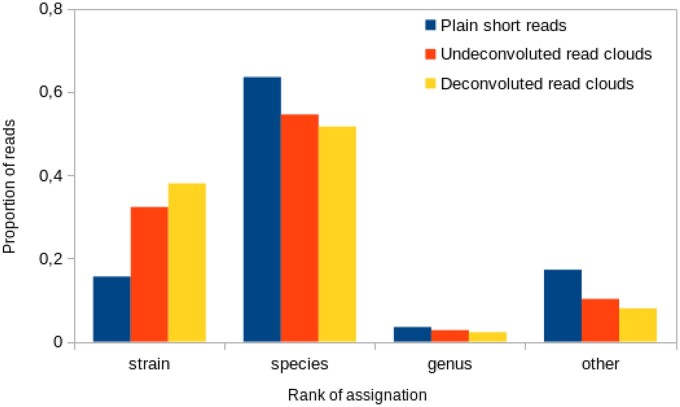
Proportion of reads assigned to each rank in the ATCC dataset. The three columns correspond to ignoring barcode information, using raw barcode information and using deconvolved barcode information

We implemented this strategy in a small, freely available script (github.com/RolandFaure/cloudClassifier), compatible with any classifier. The reads in the ATCC 10× dataset were classified by taxonomy using Kraken2 (Wood *et al.*, 2019), a popular tool. Read assignment was then enhanced using either non-deconvoluted read clouds or QD deconvoluted read clouds. The result is displayed in Figure 7. Mis-attributed reads accounted for <1% of the reads in all cases and are not shown. The use of deconvolved read clouds provides strain-resolved taxonomic assignment for significantly more reads than non-deconvolved read clouds.

Genome assembly and scaffolding are other classic applications of linked-read technologies. We expect both of these applications to be enhanced by deconvolution. Assemblers [e.g. cloudSpades (Tolstoganov *et al.*, 2019) and Supernova2.0 (Visendi, 2022)] and scaffolders [e.g. ARCS (Yeo *et al.*, 2018) and ARKS (Coombe *et al.*, 2018)] link draft contigs based on the number of barcodes they share. If unlucky, two contigs may contain multiple pairs of fragments sharing the same barcode. This could confuse the assembler/scaffolder. This problem will be largely mitigated if the reads have been deconvoluted beforehand.

## 5. Discussion

We presented QD, a new software addressing the problem of barcode deconvolution. Based on the same theoretical background as Minerva, it introduces a clustering step in the algorithm, where Minerva only computed connected components. In addition, efforts have been made to make QD fast and scalable. Today, QD outperforms all other reference-free deconvolution tools in terms of speed and accuracy. We show that it is now possible to deconvolve datasets from single species with complex and repetitive genomes.

The priority to extend this work would be to re-think the index structure to reduce RAM usage. Indeed, the RAM usage went over 1000G on the *Drosophila* and the metagenome datasets, which contained respectively 33 and 111 Gb. Bigger datasets could easily be imagined: for example, a 50-fold coverage of a diploid human would generate ∼300 Gb of data.

## Supplementary Material

vbac068_Supplementary_Data

## Data Availability

The biological data underlying this article are available in SRA at www.ncbi.nlm.nih.gov/sra, and can be accessed with accession numbers SRR14577235 (ATCC loopseq dataset) and SRR12283286 (ATCC 10X dataset). The simulated and drosophila datasets will be shared on request to the corresponding author.
