# Supplementary material for: QuickDeconvolution: fast and scalable deconvolution of linked-read sequencing data
Source: Bioinform Adv. 2022 Sep 26;2(1):vbac068. doi: 10.1093/bioadv/vbac068 (PMC9710601; doi:10.1093/bioadv/vbac068)
Supplement: vbac068_Supplementary_Data [file vbac068_supplementary_data.zip › Article_QuickDeconvolution_supplementaryMaterial.pdf]

Bioinformatics advances, 2022, pp. 1–1

doi: DOI HERE
Advance Access Publication Date: Day Month Year
Paper

PAPER

QuickDeconvolution: fast and scalable deconvolution of linked reads sequencing data - Supplementary material

Roland Faure[1,*] and Dominique Lavenier[1]

[1]Univ. Rennes, Inria RBA, CNRS UMR 6074, Rennes, France
[*]Corresponding author. roland.faure@irisa.fr
FOR PUBLISHER ONLY Received on Date Month Year; revised on Date Month Year; accepted on Date Month Year

Supplementary figures

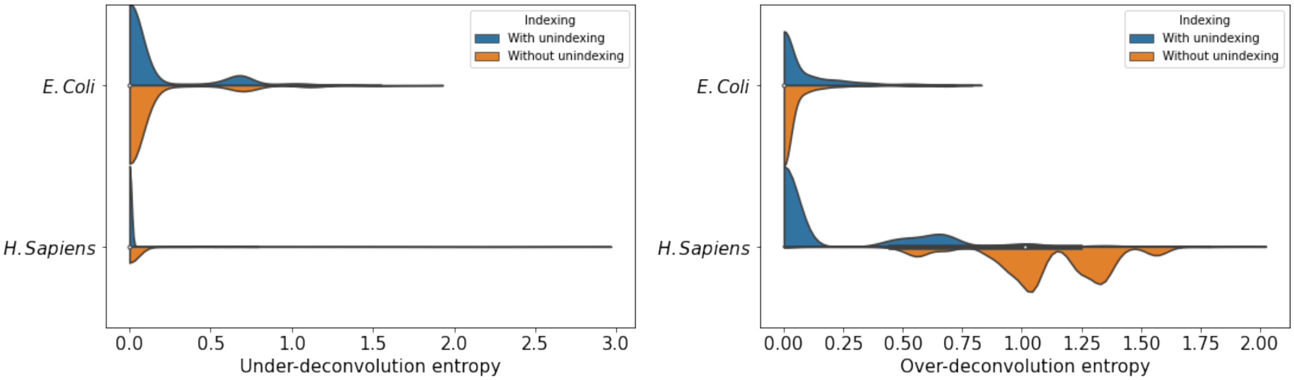

Figure S 1. Evaluation of the impact of un-indexing k-mers present twice as many times as average. Under-deconvolution and over-deconvolution entropies have been measured for the *E. coli* and *H. sapiens* datasets, both when un-indexing k-mers and leaving all k-mers indexed. While the deconvolution quality is similar on *E. coli* (with un-indexing, the reads are slightly less under-deconvoluted but slightly more over-deconvoluted), the difference is blatant on the repeat-rich chromosome 1 of *Homo sapiens*.

© The Author 2022. Published by Oxford University Press. All rights reserved. For permissions, please e-mail: journals.permissions@oup.com
